# Summary of best evidence for targeted body temperature management in patients with severe neurological illness

**DOI:** 10.3389/fmed.2026.1781153

**Published:** 2026-03-30

**Authors:** Dan Zhang, Fengmei Li, Dan Wen, Zhi Zeng, Fenglin Yan, Haiyan He, Xiuru Yang

**Affiliations:** Department of Intensive Care Unit, Mianyang Central Hospital, Affiliated with the School of Medicine, University of Electronic Science and Technology of China, Mianyang, Sichuan, China

**Keywords:** evidence-based nursing, hypothermia, ICU, neurocritical care, targeted temperature management

## Abstract

**Background:**

To retrieve, synthesise, and evaluate the best practice evidence for targeted temperature management in neurocritical care patients.

**Methods:**

A comprehensive search was conducted following the “6S” evidence evaluation framework, utilizing databases such as BMJ Best Practice, UpToDate, JBI Database of Evidence-Based Health Care (Australia), National Guideline Clearinghouse, Cochrane Library, China Biology Medicine disc, Wanfang Data, and China National Knowledge Internet. The search focused on high-quality resources related to targeted temperature management in neurocritical care, including clinical practice decisions, professional guidelines, evidence summaries, and expert consensuses. Two independent researchers conducted the quality assessment and evidence extraction.

**Results:**

Twenty documents were included, comprising one clinical decision-making article, seven guidelines, six expert consensus statements, four systematic reviews, and two evidence summaries. These documents yielded 27 best-practice pieces of evidence for targeted temperature management in neurocritical care patients, spanning six aspects: preparation, initiation, maintenance, complication management, rewarming, and prognosis management.

**Conclusion:**

The best evidence for targeted temperature management in neurocritical care patients, constructed through an evidence-based approach, provides an optimal basis for decision-making by healthcare professionals.

## Introduction

Severe neurological diseases are serious conditions affecting the brain, spinal cord, or neuromuscular system (1). These diseases, which include cerebral haemorrhage, head injury, severe stroke, and severe cerebrovascular disease, are characterised by many complications, high disability rates, and high mortality rates (2, 3). Targeted temperature management (TTM) (4) is the rapid adjustment of a patient’s body temperature to a preset level by physical and pharmacological means, and the gradual return to normal body temperature after maintenance. It includes hypothermia, controlled room temperature therapy, and fever therapy. TTM is an effective method of brain protection that can reduce inflammation and release of excitotoxic substances and improve the neurological prognosis of patients (5). It is mainly used in patients with cardiac arrest (6, 7) and is also recommended for the treatment of various brain injuries such as acute haemorrhagic and ischaemic stroke and head injury in multinational medical guidelines (8–11). Studies have reported TTM application rates between 20–69%, with some rising as high as 90% (12, 13). However, in China, the application rate is only 6.3% (14). In TTM practice, nurses, as core performers, have a positive impact on optimising patient outcomes and prognoses, which cannot be ignored (15). Several surveys have shown (16–18) that a low level of awareness about TTM among domestic nursing staff may increase the risk of patient-related complication rates (19). Evidence-based nursing interventions can effectively promote the recovery of neurological function and improve the quality of life of patients receiving neurocritical care (20). The aim of this study was to extensively search for and evaluate the latest evidence in the field of neurocritical care TTM, both domestically and internationally, and to form a summary of the best evidence through systematic integration. This will provide a solid basis for clinical decision-making and help construct a standardised and scientific clinical care pathway.

## Information and methods

### Problem establishment

The evidence-based question establishment tool PIPOST (21) of the Center for Evidence-Based Nursing of Fudan University was used to form evidence-based practice questions: (1) P (Population): adult patients with severe neurological illness (e.g., traumatic brain injury, cardiac arrest, stroke); (2) I (Intervention): clinical practices related to targeted temperature management, encompassing assessment of indications and contraindications, temperature monitoring methods, target temperature setting, cooling techniques, maintenance duration, rewarming strategies, and complication prevention nursing protocols; (3) P (Professionals): neurocritical care physicians, emergency department physicians, and intensive care unit (ICU) nurses; (4) O (Outcomes): effectiveness indicators: intracranial pressure control rate, neurological functional outcome scores (e.g., Glasgow Outcome Scale, GOS), mortality, ICU length of stay. Safety indicators: hypothermia-associated complications (arrhythmias, coagulation disorders), shivering incidence, secondary infection risk; (5) S (Setting): emergency department, neurocritical care unit (NICU), and general intensive care unit (ICU); (6) T (Evidence types): clinical decision protocols, evidence summaries, systematic reviews, guidelines, and expert consensus. The protocol for this best evidence summary was registered in the Center for Evidence-Based Nursing of Fudan University (Registration Number: ES20269970).

### Search strategy

Following the framework of the “6S” pyramid of evidence model, top-down searches were conducted in authoritative databases worldwide: BMJ Best Practice, UpToDate, International Guidelines Collaboration, Australian JBI Centre for Evidence-Based Health Care database, National Institute for Health and Clinical Excellence (NICE), Scottish Intercollegiate Guidelines Network (SIGN), National Guidelines Collection (NGC), Cochrane Library, CINAHL, Pubmed, New Zealand Guidelines Collaboration, China Medical Pulse Guidelines, China National Knowledge Infrastructure (CNKI), Wanfang Database, and China Biomedical Literature Database (CBM). The search terms were as follows: brain damage/brain injuries/stroke/cerebral hemorrhage/hypothermia, induced/targeted temperature management/therapeutic hypothermia/mild hypothermia/therapeutic normothermia/controlled normothermia/fever. The terms were adapted to the specific syntax of each database. An example of the full search strategy used in PubMed is provided below:

# 1 (“hypothermia, induced”[Mesh]) OR (targeted temperature management OR hypothermia* OR therapeutic hypothermia OR cooling OR rewarming OR controlled normothermia).
# 2 (“Brain damage”[Mesh]) OR (Brain Injuries OR stroke OR Cerebral Hemorrhage).
# 3 (guideline[Title/Abstract] OR best practice[Title/Abstract] OR expert consensus[All Fields] OR meta-analysis[Title/Abstract] OR systematic review[Title/Abstract] OR evidence[Title/Abstract]).
# 4 = #1 AND #2 AND #3.

The search period was from January 01, 2014 to December 31, 2025.

### Selection and exclusion criteria

Inclusion criteria: (1) critically ill neurological patients aged ≥18 years; (2) the content covers the assessment of target temperature management, interventions, complications, and prognosis; (3) the outcome measures focused on the incidence of complications and the recovery of neurological function; (4) limited to guidelines, systematic reviews, expert consensus, best practice cases, and evidence summaries published in the past 10 years that are closely related to the management of target temperature for adult neurocritical illness; and (5) the language is Chinese or English. The objective was to summarize pre-appraised, synthesized evidence to directly inform practice. Therefore, primary research studies (e.g., randomized controlled trials, cohort studies) were excluded at the screening stage. Exclusion criteria: (1) The types of literature were guideline interpretation, single case report, research plan or draft protocol; (2) the full text is not available; and (3) rated C in the quality evaluation, had significant methodological deficiencies, or incomplete data.

### Literature evaluation criteria

(1) Clinical practice guidelines were appraised using the AGREE II tool (2012 updated version) (22), which evaluates six domains on a 7-point scale.
(2) The methodological quality of systematic reviews was assessed using the 11-item AMSTAR checklist. Each item was rated as “yes,” “no,” “unclear,” or “not applicable.”
(3) The 2016 JBI expert consensus evaluation criteria for evidence-based health care centers were used to evaluate expert consensus.
(4) Best practices and evidence summary: Select the corresponding evaluation tools of JBI according to the type of original evidence literature. Original evidence underwent quality assessment using corresponding JBI critical appraisal tools, with each tool’s signaling questions rated as “yes,” “no,” “unclear,” or “not applicable.”

### Literature quality evaluation process

Quality evaluation was conducted independently by two researchers with backgrounds in evidence-based nursing training at Fudan University. Any disagreements in quality assessment or study selection were first discussed between the two researchers to reach a consensus. If a consensus could not be reached, a third senior researcher or a member of the expert panel was consulted for a final decision. Specific inclusion principles were followed when dealing with conflicting conclusions of evidence: prioritising evidence-based evidence first, high-quality evidence second, and recently published authoritative literature last.

### Summary of evidence

Two researchers with evidence-based nursing training backgrounds and more than 5 years of ICU experience used content analysis to refine the key content of target temperature management for neurocritical illness. Evidence recommendation grading was based on the FAME framework (feasibility, appropriateness, meaningfulness, effectiveness). Recommendations achieving threshold scores in ≥3 FAME domains were classified as Grade A (strong recommendation). Those meeting 1–2 criteria or exhibiting significant implementation barriers were designated Grade B (weak recommendation).

## Results

### Literature search results

**Figure 1:**
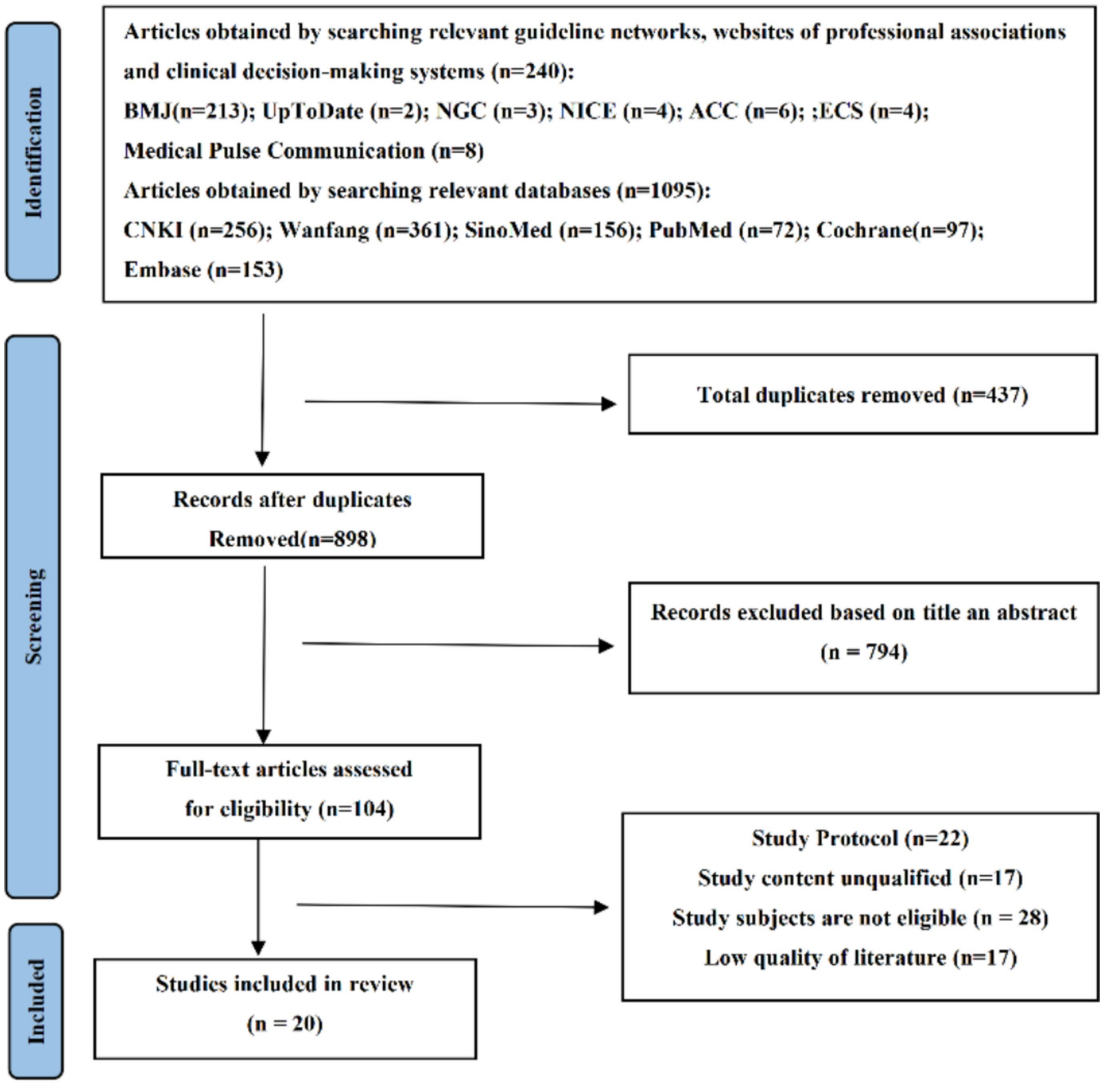
Flow chart of literature screening.

This study included a total of twenty articles, including one clinical decision-making (23), seven guidelines (6–9, 24–26), six expert consensuses (10, 27–31), four systematic reviews (32–35), and two evidence summaries (19, 36). The literature selection process is illustrated in Figure 1, and the general characteristics of the included literature are presented in Table 1.

**Table 1:** Basic characteristics of the included literature.

Included literature	Year	Literature source	Literature topics	Literature type
Madden (8)	2017	Evidence-based guidelines from the Society for Neurocritical Care Nursing	Implementing target temperature management: evidence-based guidelines from the society for neurocritical care nursing	Guideline
Sandroni (6)	2022	European Resuscitation Council and European Society of Critical Care Medicine	Guidelines for temperature control after cardiac arrest in adults	Guideline
Nolan (7)	2021	Critical Care Medicine	Guidelines 2021 from the European Resuscitation Council and the European Society of Intensive Care Medicine: post-resuscitation care	Guideline
Cariou (9)	2017	Pubmed	Targeted temperature management in the ICU: guidelines from a French expert panel	Guideline
Gai (19)	2023	CNKI	Summary of the best evidence for target temperature management in patients with brain injury in the ICU	Summary of the evidence
Kyriazopoulou (32)	2020	Cochrane Library	Sinus bradycardia during targeted temperature management: a systematic review and meta-analysis	Systematic review
Crombez (33)	2017	PubMed	Effect of target temperature management on drug pharmacokinetics administered during and after cardiac arrest	Systematic review
Andrews (10)	2018	PubMed	Target temperature management in patients with intracerebral hemorrhage, subarachnoid hemorrhage, and acute ischemic stroke	Expert consensus
Geocadin (25)	2019	CNKI	Expert consensus on the clinical practice of target body temperature management in adult patients with acute and critical brain injury	Expert consensus
Neurocritical Care Expert Committee of the Neurosurgery Branch of the Chinese Medical Doctor Association (28)	2022	CNKI	Chinese expert consensus on target temperature management for neurocritical illness (2022 edition)	Expert consensus
Emergency Physician Branch of Chinese Medical Doctor Association (27)	2020	CNKI	Chinese expert consensus on hypothermia brain protection	Expert consensus
Fiorilli (23)	2022	UpToDate	ICU management of adult intubated patients after cardiac arrest	Clinical decision-making
Cardiac Critical Care & Brain Protection Group (29)	2019	American Academy of Neurology	Reduces brain injury after CPR in adults	Guideline
Sandroni (35)	2022	PubMed	Using brain biomarkers to predict neurological prognosis after cardiac arrest	Systematic review
Zhang (36)	2020	CNKI	Summary of the best evidence for target temperature management in patients with cardiac arrest	Summary of the evidence
Donnino (26)	2015	International Liaison Committee on Resuscitation/American Heart Association	Temperature management after cardiac arrest	Guideline
Chiu (30)	2021	PubMed	Care after cardiac arrest and target temperature management	Expert consensus
Hoiland (34)	2022	PubMed	Predicts neurological prognosis in comatose patients with cardiac arrest	Systematic review
Emergency Physicians’ Branch of Chinese Medical Association (31)	2025	CNKI	Chinese expert consensus on pre-hospital and emergency diagnosis and treatment of adult traumatic brain injury	Expert consensus
Nolan (24)	2025	PubMed	European Resuscitation Council and European Society of Intensive Care Medicine guidelines 2025: post-resuscitation care	Guideline

CNKI, China National Knowledge Infrastructure.

### Quality evaluation results of the literature

(1) Quality evaluation of guidelines: A total of seven guidelines (6–9, 24–26) were included in this study, and the overall quality was high. The evaluation results are shown in Table 2.
(2) Quality evaluation results of the systematic review: A total of four systematic reviews (32–35) were included in this study, with an overall high quality (Table 3).
(3) Quality evaluation results of expert consensus: A total of six expert consensuses (10, 27–31) were included in this study, including two international expert consensuses and four domestic expert consensuses; all six evaluation criteria were “yes,” with high overall quality (Table 4).
(4) Quality evaluation results of clinical decision-making and evidence summarization: A total of one clinical decision (23) and two evidence summaries (19, 36) were included in this study, with the extracted evidence obtained from two guidelines, four systematic reviews, and five expert consensuses, all of which were included in this study; all of them were of high quality.

**Table 2:** Results of guideline quality evaluation.

Included literature	Percentage of standardization by domain						≥60% of the number of fields	≥30% of the number of fields	Recommended level
	Scope and purpose	Participants	Rigor of formulation	Clarity of presentation	Usefulness of the guide	Editorial independence			
Madden (8)	100	60.4	87.5	90.4	66.7	100	6	6	A
Sandroni (6)	100	95.2	83.9	93.8	54.2	83.3	5	6	B
Nolan (7)	95.2	96.4	92.8	87.5	85.7	66.7	6	6	A
Cariou (9)	76.4	82.5	93.4	78.7	89.4	73.1	6	6	A
Nolan (24)	96.1	95.8	94.7	90.2	89.6	79.3	6	6	A
Geocadin (25)	98.8	61.9	73.2	87.5	54.2	87.5	5	6	B
Donnino (26)	83.3	90.4	72.9	94.4	41.7	81.2	5	6	B

**Table 3:** Quality evaluation results of the systematic review.

Items	Kyriazopoulou (32)	Crombez (33)	Hoiland (34)	Sandroni (35)
1. Did the research questions and inclusion criteria for the review include the components of PICO?	Yes	Yes	Yes	Yes
2. Did the report of the review contain an explicit statement that the review methods were established prior to the conduct of the review and did the report justify any significant deviations from the protocol?	Yes	Yes	Yes	Yes
3. Did the review authors explain their selection of the study designs for inclusion in the review?	Yes	Yes	Yes	Yes
4. Did the review authors use a comprehensive literature search strategy?	Yes	Yes	Yes	Yes
5. Did the review authors perform study selection in duplicate?	Yes	Yes	Yes	Yes
6. Did the review authors perform data extraction in duplicate?	Yes	Yes	Yes	Yes
7. Did the review authors provide a list of excluded studies and justify the exclusions?	Yes	Yes	Yes	Yes
8. Did the review authors describe the included studies in adequate detail?	Yes	Yes	Yes	Yes
9. Did the review authors use a satisfactory technique for assessing the risk of bias (RoB) in individual studies that were included in the review?	Yes	Yes	Yes	Yes
10. Did the review authors report on the sources of funding for the studies included in the review?	No	No	No	No
11. If meta-analysis was performed did the review authors use appropriate methods for statistical combination of results?	Yes	Yes	Yes	No
12. If meta-analysis was performed, did the review authors assess the potential impact of RoB in individual studies on the results of the meta-analysis or other evidence synthesis?	Yes	Yes	Yes	No
13. Did the review authors account for RoB in individual studies when interpreting/ discussing the results of the review?	Yes	Yes	Yes	Yes
14. Did the review authors provide a satisfactory explanation for, and discussion of, any heterogeneity observed in the results of the review?	Yes	Yes	Yes	Yes
15. If they performed quantitative synthesis did the review authors carry out an adequate investigation of publication bias (small study bias) and discuss its likely impact on the results of the review?	Yes	Yes	Yes	No
16. Did the review authors report any potential sources of conflict of interest, including any funding they received for conducting the review?	No	No	No	No

**Table 4:** Quality evaluation results of expert consensus.

Items	Andrews (10)	Emergency Physician Branch of Chinese Medical Doctor Association (27)	Neurocritical Care Expert Committee of the Neurosurgery Branch of the Chinese Medical Doctor Association (28)	Cardiac Critical Care & Brain Protection Group (29)	Chiu (30)	Emergency Physicians’ Branch of Chinese Medical Association (31)
(1) Whether a clear source of point of view is presented?	Yes	Yes	Yes	Yes	Yes	Yes
(2) Whether the opinion comes from influential experts in the field?	Yes	Yes	Yes	Yes	Yes	Yes
(3) Whether the arguments presented are centered on the interests of the people involved in the study?	Yes	Yes	Yes	Yes	Yes	Yes
(4) Whether the stated conclusions are based on the results of the analysis?	Yes	Yes	Yes	Yes	Yes	Yes
(5) Whether other existing literature has been consulted and accurately indexed?	Yes	Yes	Yes	Yes	Yes	Yes
(6) Whether there are any inconsistencies between the points presented and previous literature?	Yes	Yes	Yes	Yes	Yes	Yes

### Summary of evidence results

This study summarises the best evidence of 27 target body temperature management practices in patients with severe neurological illnesses in six aspects: preparation, initiation, maintenance, complication management, rewarming, and prognosis management (Table 5).

**Table 5:** Summary of the best evidence on target temperature management practices for neurocritical illness.

Theme of evidence	Content of evidence	Level of recommendation
Preparation	1. Invasive blood pressure monitoring should be continued, hemodynamic stability should be stabilized, or low-dose vasoactive drugs should be given to maintain blood pressure (19)	B
	2. Establish effective central venous access (36)	B
	3. Establish an artificial airway and provide mechanically assisted ventilation (23)	B
	4. It is recommended to place a nasogastric tube or nasoenteric tube (28)	A
	5. Before starting TTM, analgesic and sedative drugs should be used to prevent chills, but the patient’s cough and spontaneous breathing should be preserved, and the Richmond agitation-sedation score was −4 point (6)	A
	6. It is recommended that patients treated with TTM continuously monitor their core body temperature, and based on the advantages of non-invasive and easy operation, esophageal temperature is the first choice, followed by bladder temperature, and body surface temperature can be monitored when implementing normal body temperature control (32)	A
	7. Cooling equipment: Preferential selection of new body surface cooling technology with temperature feedback control device or intravascular cooling technology for TTM. If you do not have the conditions, you can also choose traditional body surface cooling techniques (ordinary ice blankets, ice caps, ice packs) for cooling, but you need to monitor the core temperature more closely (28)	A
Induction	8. With the goal of maintaining a normal body temperature (36–37 °C), actively prevent fever (≤37.5 °C) (24, 31). For patients with refractory increased ICP, maintaining the core temperature at 32 to 35 °C may be considered (31)	A
	9. Target temperature maintenance time: 24–72 h for patients with acute ischemic stroke and large-scale cerebral infarction, and 3–5 days for patients with hemorrhagic stroke (28). Patients with refractory increased ICP may consider long-term mild hypothermia treatment (for more than 5 days) (31)	A
	10. Patients with acute head injury, ischemic stroke and hemorrhagic stroke should start TTM treatment as soon as possible (6–72 h) (7, 8, 19). For patients with traumatic brain injury, the primary goal in the initial management is the prevention of fever and maintenance of normothermia (31)	A
Maintenance	11. Real-time monitoring of vital signs, measurement of central venous pressure, intracranial pressure, cerebral perfusion pressure, skin temperature, core temperature (esophagus, bladder or rectum), and observation of pupil changes (8, 34)	B
	12. Monitor blood gas analysis every 1–2 h, and adjust the target pH value to be 7.35–7.45, arterial carbon dioxide partial pressure to be 35–40 mmHg, arterial blood oxygen partial pressure to be 80-120 mmHg, and potassium ion concentration to be 3.5–5.3 mmol/L (8, 9)	B
	13. Maintain blood glucose level at 7.8–10.0 mmol/L (adjust the frequency of blood glucose testing according to blood glucose level) (7, 25)	B
	14. Regular review of cranial CT, regular neurological examination, continuous or intermittent EEG, cerebral oxygen and cerebral blood flow monitoring (28, 29)	B
	15. It is recommended to use electromyography to continuously monitor the occurrence of chills, and the severity of chills can also be assessed using the chills assessment scale (19, 32)	A
	16. Enteral nutrition should be started at a low rate early (within 24–48 h after entering the intensive care unit), and the feeding rate should be adjusted according to the gastrointestinal situation (27, 36)	B
	17. When enteral nutrition exceeds the 7-day energy target and cannot be reached (< 60% energy target), parenteral nutrition supplementation is recommended (10)	B
	18. Nasogastric tube feeding is preferred for enteral nutrition, and post-pyloric feeding can be used for patients who are intolerant to nasogastric tube feeding and have a high risk of aspiration (23, 25)	A
	19. Low-dose (75%) enteral nutrition can be given during hypothermia treatment, and the dose can be gradually increased after rewarming (28, 33)	B
Complication management	20. It is recommended to monitor intra-abdominal pressure (IAP) and gastric residual volume (GRV) every 4–6 h. When the IAP is 12–15 mmHg, routine enteral nutrition can be continued; When the IAP is 16–20 mmHg, low-speed nourishing feeding should be used. When the IAP >20 mmHg, enteral nutrition should be suspended. Low-speed enteral feeds can be given when the GRV exceeds 200 mL, and enteral nutrition should be suspended when the GRV >500 mL (19, 26)	A
	21. During the implementation of enteral nutrition, gastrointestinal motility drugs, laxatives or enemas can be given, abdominal insulation can be given, and if necessary, the rehabilitation department can be asked to implement gastrointestinal electrical stimulation (30, 32, 33)	B
	22. Strengthen the monitoring of arrhythmias, such as sinus bradycardia, ventricular tachycardia, atrial fibrillation, ventricular fibrillation, and QT interval prolongation (8, 32)	A
Rewarming	23. It is recommended to control the rewarming of patients treated with TTM, and the rate of rewarming is 0.25–0.5°C/h (7, 27)	A
	24. After rewarming, the core body temperature should be controlled below 37.5 °C and maintained at least 72 h after resuscitation (19, 29)	A
	25. In the rewarming stage, the frequency of blood gas analysis and electrolyte examination is once every 2–4 h (6, 8)	B
Prognostic management	26. Patients with severe neurological illness are recommended to use the Glasgow Coma Scale (GCS), activities of daily living (Barthel index), modified Rankin scale, and National Institutes of Health Stroke Scale (NIHSS) to assess neurological prognosis after 6 months to guide treatment strategies. Furthermore, the Montreal Cognitive Assessment (MoCA) is recommended for evaluating long-term cognitive function, and the SF-36 (Medical Outcomes Study 36-Item Short-Form Health Survey) quality of life scale is suggested for assessing patients’ overall quality of life (28, 29, 35)	A
	27. It is recommended to follow up the short-term (≤1 month) and long-term (≥3 months) mortality rate of patients to evaluate the efficacy of TTM (7, 19)	B

TTM, targeted temperature management; EEG, electroencephalography; IAP, intra-abdominal pressure; GRV, gastric residual volume; GCS, Glasgow Coma Scale; NIHSS, National Institutes of Health Stroke Scale; MoCA, Montreal Cognitive Assessment; SF-36, Medical Outcomes Study 36-Item Short-Form Health Survey.

## Discussion

### Preparations should be made before initiating target temperature management

Evidence 1 to 7 summarises the evidence in the preparatory phase for target temperature management in patients with neurological diseases, including access establishment, temperature monitoring, and cryotherapy selection. Hypothermia often involves the use of cryogenic devices and sedative analgesics to slow metabolism and reduce oxygen consumption; however, it also inhibits cough and ciliary activity and weakens airway protection and diaphragm function (37). The available evidence (38) recommends that deep sedation be provided during target temperature management in patients with severe neurological disease. In this context, the Richmond agitation-sedation score can reach −4 points, the depth of anaesthesia ranges from 30 to 50, and the patient’s cough and spontaneous breathing are preserved to maintain lung function and respiratory muscle strength and reduce complications such as ventilator-associated pneumonia. In addition, patients are affected by hypothermia, analgesia and sedation, etc., and are prone to adverse reactions such as blood pressure fluctuations, “cold diuresis” (hypovolemia, hypokalaemia, hypomagnesemia, and hypophosphatemia), and feeding intolerance. Invasive blood pressure measurement facilitates real-time monitoring and blood specimen collection. Central venous access allows for timely infusion of analgesics, sedatives, and vasoactive drugs. Early placement of a nasogastric or nasoenteric tube reduces gastric retention or aspiration due to decreased gastrointestinal peristalsis (38). Core body temperature monitoring is essential for the management and prognosis of patients with severe neurological illnesses (39) and should be monitored continuously or at least hourly (40). The brain is the preferred site for temperature measurement; however given its safety and clinical feasibility, much evidence recommends monitoring sites such as the oesophagus, bladder, and rectum (6, 33). Bladder temperature is affected by fluctuations in urine output and rectal thermometry has a slow response (41). Medical staff must comprehensively consider the monitoring results and flexibly choose the best temperature measurement scheme according to clinical practice.

### Clarify the management objectives of the induction stage

The management objectives for temperature control in neurocritical care have evolved significantly, as reflected in the latest evidence. The 2025 ERC-ESICM guidelines (24) introduce a pivotal shift in post-cardiac arrest care, moving the emphasis from “targeted temperature management” to “temperature control” with the primary goal of actively preventing fever (core temperature ≤37.5 °C). A key operational change is the recommendation not to actively rewarm patients who are already mildly hypothermic (32–36 °C) at the restoration of spontaneous circulation. This shift is directly supported by the landmark TTM2 trial (42), which found that for comatose adults after out-of-hospital cardiac arrest, induced hypothermia (33 °C) provided no survival or neurological benefit over strict normothermia (≤37.5 °C) with fever prevention. Consequently, the contemporary paradigm prioritizes rigorous fever avoidance over mandating a specific low temperature.

This principle of fever prevention as a critical, universal goal extends across neurocritical care but is applied with condition-specific nuance. The 2025 Chinese expert consensus on traumatic brain injury exemplifies this by establishing maintaining normothermia (36–37 °C) as the primary and fundamental objective to prevent secondary injury (31). It positions therapeutic hypothermia (32–35 °C) strictly as a second-tier intervention reserved for cases of refractory intracranial hypertension. For other neurological conditions such as stroke, subarachnoid haemorrhage, and hepatic encephalopathy, the recommended target body temperature remains at 33.0–36.0 °C when hypothermia therapy is indicated (25), reflecting the more heterogeneous and less definitive evidence base for actively inducing hypothermia versus maintaining normothermia in these specific populations. Regarding practical application, although the optimal time window for initiation may vary, management should commence as soon as possible after illness onset to mitigate cerebral oedema and inflammation, with evidence supporting initiation within 6–72 h for conditions like acute head injury and stroke (16, 17). Maintenance durations are also condition-dependent: at least 72 h of fever prevention for post-cardiac arrest care, 24–72 h for acute ischemic stroke, and 3–5 days for hemorrhagic stroke (10).

### During the target body temperature maintenance period, monitoring should be strengthened and nutritional support should be provided

Evidence 11 to 19 summarises the key points of monitoring during target temperature management. In the management of target body temperature, comprehensive monitoring and control of key indicators—including vital signs, intracranial pressure, blood gas analysis, blood glucose, and the nervous system—is required. Studies have shown that (41) for every 1 °C decrease in core temperature, the cerebral metabolic rate decreases by 6–7%. Cerebral blood flow monitoring can help detect abnormal changes in cerebral blood flow in time and hence, prevent or alleviate brain damage caused by cerebral ischaemia or hyperperfusion. Chills are reported in 40% of patients during TTM (43), which, if left uncontrolled, can increase metabolism, oxygen consumption, energy expenditure, and carbon dioxide production (44), counteracting the brain-protective effects of TTM. Electromyography can continue to objectively monitor the occurrence of chills; however, specialised equipment is required for this. The chills assessment scale (45) can be used to clinically assess the severity of chills. Enhanced surface warmth reduces cold irritation and is effective in preventing shivering in intravascular hypothermia but is less effective in surface cooling (28). Sedation is an important means of suppressing chills (28), which can be relieved or eliminated by drugs such as acetaminophen, magnesium, sedative-hypnotics, narcotic analgesics, and neuromuscular blockers (10). Hypothermia slows down drug metabolism and excretion; therefore, the dose and timing of drug administration should be monitored (28). Available evidence suggests (46) that enteral nutrition should be initiated at a low rate as early as possible, within 24–48 h of ICU admission, with feeding rates adjusted according to gastrointestinal tolerance. A nasogastric tube is preferred, and retropyloric feeding should be considered in patients at high risk of feeding intolerance or aspiration (47). If the 60% energy goal is not achieved after 7 days of enteral nutrition, parenteral supplementation is recommended (47).

### Strengthen complication management

Evidence 20 to 22 summarises the key points for preventing complications during target temperature management. Patients with hyperthermia have increased cerebral blood flow and decreased gastrointestinal perfusion, disrupting the gastrointestinal mucosal barrier and resulting in slowed gastrointestinal motility and decreased intestinal absorption (48). Moreover, drugs and low temperature can directly or indirectly affect gastrointestinal function, resulting in decreased gastrointestinal peristalsis and dysbiosis. Intra-abdominal pressure and gastric residual volume should be monitored at regular intervals, at least once daily, every 4–6 h after abdominal distension occurs (49). Gastric residual volume can be monitored using syringe aspiration or gastric ultrasound to ensure that the gastrointestinal tract’s status is assessed in a timely manner (50). The available evidence recommends that for patients with decreased abdominal temperature, thermal insulation measures such as thermal blankets, hot water bottles, etc., can be used to increase abdominal temperature and promote gastrointestinal peristalsis and digestive function. If necessary, gastrointestinal motility drugs or gastrointestinal electrical stimulation can be used to promote intestinal peristalsis. During the process of target body temperature management, especially when therapeutic hypothermia is administered, patients may be affected by a variety of factors such as thermoregulatory centres, drug use, and inadequate nutrient intake, resulting in electrolyte imbalances, especially hypernatremia, hyponatremia, and hypokalaemia (51). This results in abnormal myocardial cell excitability and thus induces arrhythmias. In the management of target body temperature, electrolyte levels should be monitored regularly to correct abnormalities and prevent arrhythmias.

### Pay attention to rewarming and prognosis assessment

Rapid rewarming may result in systemic vasodilation and hypotension, which may reduce cerebral blood flow, cause cerebral oedema, and increase intracranial pressure, negating the benefits of TTM. The available evidence (51) suggests that the target temperature for rewarming is 36.0–37.5 °C and that the rate of rewarming should not exceed 0.1–0.25 °C/h for at least 72 h. After the TTM device is removed, healthcare providers need to continuously monitor vital signs and temperature changes and maintain the core temperature of critically ill neurological patients within the target range using antipyretic analgesic drugs and assisted cooling (intravenous cold saline, ice packs, and plain ice blankets). For prognostic assessment, it is recommended that the Glasgow Coma Scale, Barthel index, modified Rankin scale, and NIHSS be used to comprehensively assess neurological prognosis after 6 months (35). Furthermore, the Montreal Cognitive Assessment (MoCA) is recommended for evaluating long-term cognitive function, and the SF-36 (Medical Outcomes Study 36-Item Short-Form Health Survey) quality of life scale is suggested for assessing patients’ overall quality of life (35). At the same time, the short-term (≤1 month) and long-term (≥3 months) mortality rates of patients were recorded at the same time to objectively evaluate the efficacy of TTM therapy.

## Limitations

This evidence summary incorporated systematic reviews, clinical guidelines, and expert consensus statements, each with unique strengths and limitations. Systematic reviews provide rigorous syntheses of primary research but may be limited by study heterogeneity and publication bias. Clinical guidelines offer structured recommendations but can suffer from slow updates and institutional influences. Expert consensus statements, while valuable for rapid guidance, are inherently subjective and lack robust empirical support. Additionally, while we employed a comprehensive search strategy following the “6S” model, it was limited to Chinese and English publications. This may have introduced language bias and led to the omission of relevant evidence published in other languages. Furthermore, despite searching multiple databases, it is possible that some systematic reviews meeting our inclusion criteria were not indexed or were unpublished. To mitigate the impact of these limitations and any single evidence source being missed, we prioritized the integration and synthesis of recommendations from multiple high-quality, synthesized source types. The conclusions of this summary are therefore based on the concordance of evidence across these diverse sources, which strengthens their reliability.

## Conclusion

This study synthesised the latest and best practice evidence on target temperature management strategies in the field of neurocritical care, both domestically and internationally. The overall quality was high, and can provide a basis for clinical decision-making. Addressing these priorities will establish a robust scientific foundation for individualized nursing practice.
